# Draft Genome Sequence of an Enterotoxigenic Escherichia coli Strain Carrying Genes for Colonization Surface Antigen 13 and a Heat-Labile Toxin

**DOI:** 10.1128/mra.00416-22

**Published:** 2022-09-12

**Authors:** Samuel M. Njoroge, Laure F. Madé, Astrid von Mentzer, Benard W. Kulohoma, Timothy K. Kamanu, Tom T. Ouko, John Kiiru, Melissa J. Ward, Nicholas R. Thomson, Eric M. Fèvre, Mark Woolhouse, Samuel Kariuki

**Affiliations:** a University of Nairobi, Department of Biochemistry, Nairobi, Kenya; b International Livestock Research Institute, Nairobi, Kenya; c Institute of Infection, Veterinary and Ecological Sciences, University of Liverpool, Liverpool, United Kingdom; d Chalmers University of Technology, Department of Mathematical Sciences, Gothenburg, Sweden; e University of Nairobi, School of Mathematics, College of Biological and Physical Sciences, Nairobi, Kenya; f Centre for Microbiology Research, Kenya Medical Research Institute, Nairobi, Kenya; g Ministry of Public Health and Sanitation, National Public Health Laboratory Services, Nairobi, Kenya; h Nuffield Department of Clinical Medicine, University of Oxford, John Radcliffe Hospital, Oxford, United Kingdom; i Centre for Immunity, Infection, and Evolution, University of Edinburgh, Edinburgh, Scotland, United Kingdom; j Wellcome Sanger Institute, Hinxton, United Kingdom; k Usher Institute of Population Health Sciences and Informatics, University of Edinburgh, Edinburgh, Scotland, United Kingdom; l Kenya Medical Research Institute, Nairobi, Kenya; University of Maryland School of Medicine

## Abstract

Here, we report the draft genome of ESEI_597, an enterotoxigenic Escherichia coli (ETEC) strain harboring genes encoding colonization surface antigen 13 (CS13) and a heat-labile toxin. The ESEI_597 strain was isolated from an 8-month-old child living in Korogocho, Kenya, in 2013.

## ANNOUNCEMENT

Enterotoxigenic Escherichia coli (ETEC) strain ESEI_597 was isolated in 2013 from a stool sample from an 8-month-old child residing in Korogocho, an informal settlement northeast of Nairobi, Kenya. A pea-sized fecal sample was emulsified in 5 mL buffered peptone water (Oxoid, UK). Approximately 10 μL of the overnight broth inoculum was plated on MacConkey agar (Oxoid), and a biochemically identified colony was subcultured in Mueller-Hinton agar (Oxoid) before being stocked at −80°C in tryptone soy broth (Oxoid) with 15% glycerol. All incubations were performed at 37°C for 18 to 24 h. Labile toxin (LT) and colonization surface antigen 13 (CS13) conventional uniplex PCR screening was performed using previously described primers (1, 2). Genomic DNA extraction was performed using a TANBead system (Taiwan Advanced Nanotech, Inc., Taiwan). Library preparation was performed using the NEBNext Ultra DNA library preparation kit for Illumina (New England Biolabs) and standard Illumina multiplexing adapters, with minor modifications to the manufacturer’s protocol (3). Paired-end reads (150 bp long) were generated using a HiSeq2500 platform (Illumina) at the Oxford Genomics Centre (https://www.well.ox.ac.uk/ogc) and preprocessed using an automated protocol developed by the Modernising Medical Microbiology (MMM) Oxford Group. Read trimming to remove remnant adaptor sequences was performed using BBDuk, part of the BBTools package (4) (parameters: minoverlap=12, k=19, mink=12, hdist=1, ktrim=r). Kraken v0.10.6-beta (5) was used for species identification analysis against an in-house database downloaded from the NCBI Sequence Read Archive (SRA) (www.ncbi.nlm.nih.gov/sra), with an automated step for removal of contaminant reads. The remaining reads were mapped (using Stampy v1.0.23) to an E. coli reference genome (GenBank accession AE014075.1) (6). The quality of a total of 1,485,420 reads was assessed with FastQC v0.11.8 (http://www.bioinformatics.babraham.ac.uk/projects/fastqc), with all per-base-sequence quality flagged as passed.

The draft genome was assembled using SPAdes v3.12.0 with k-mers adjusted to 33, 55, and 91, and the coverage cutoff was set as auto (7). Contigs of <200 bp were removed. The assembled genome has 139 contigs, with an *N*_50_ value of 140,019 bp, a G+C content of 50.71%, and a total length of 4,937,264 bp. ABRicate v1.0.1 (https://github.com/tseemann/abricate) was used to detect acquired antimicrobial resistance (AMR) genes with ResFinder v2.1 (8). Multilocus sequence typing (MLST) was performed using MLST v2.19 (https://github.com/tseemann/mlst) and the PubMLST database (https://pubmlst.org) (9), and plasmid replicons were screened using PlasmidFinder (10). Automatic annotation was performed by the NCBI Prokaryotic Genome Annotation Pipeline (PGAP) v6.1, with manual curation of colonization factor genes. A total of 4,795 coding sequences were predicted. Default settings were used for all software unless otherwise specified.

ESEI_597 belongs to sequence type 155 (ST155) (Achtman scheme) (11); plasmid replicons IncQ1 and IncFII(pSE11) were identified, with 100% and 99.62% sequence identity, respectively. Based on the large-scale BLAST score ratio (LS-BSR), the *aat* operon, *etpBAC* operon, *eatA*, and *tleA*-like autotransporter are all absent. The *cexE* gene is present but truncated, with an LS-BSR value of 0.8893 (12). The CS13 operon (*cshABCDEFGH*) is 7,923 bp long, and LT in ESEI_597 has been assigned to allele 29.

The Kenya Medical Research Institute (KEMRI)/National Ethics Review Committee approved all procedures (protocol number 2507).

### Data availability.

The draft genome of E. coli ESEI_597 has been deposited in GenBank under the accession number JALDSW000000000. Other features of the sequence data are provided in Table 1.

**TABLE 1:** E. coli ESEI_597 features

Characteristic	Finding
Genome size (Mbp)	4,937,264
No. of contigs	139
*N*_50_ (bp)	140,019
G+C content (%)	50.7
No. of coding sequences	4,795
No. of tRNAs	82
No. of complete rRNAs	1
AMR genes (% identity)	*aadA4* (100), *bla*_TEM-1B_ (100), *dfrA21* (99.8), *sul2* (99.88), *bla*_EC-18_ (99.21)
Replicons (% identity)	IncFII(pSE11) (99.62), IncQ1 (100)
MLST (Achtman scheme) ST	155
LT position^*a*^	4654598..4655766
CS13 operon position^*a*^	3066924..3074846
MLST (Pasteur scheme) ST	21
BioProject accession no.	PRJNA802315
BioSample accession no.	SAMN26012842
SRA accession no.	SRR18056643
GenBank accession no.	JALDSW000000000

a Genes are on the reverse strand.
